# Fall detection among elderly persons using FallCNN and transfer learning models

**DOI:** 10.3389/frai.2026.1734096

**Published:** 2026-03-11

**Authors:** K. Jishnuraj, M. Vergin Raja Sarobin, Jani Anbarasi, Graceline Jasmine, Rukmani Panjanathan

**Affiliations:** School of Computer Science and Engineering, Vellore Institute of Technology, Chennai, India

**Keywords:** CNN, deep learning, fall detection, performance analysis, transfer learning model

## Abstract

According to data provided by the World Health Organization (WHO), falls are one of the major reasons for unintentional deaths or injuries in older adults. Although many fall detection methods and algorithms exist, there is no efficient artificial intelligence strategy for fall detection. Various studies have stated that Fall Detection among Elderly Persons (FDEP) provides the possibility of developing an efficient and cost-effective way to tackle this problem. This study generated a signal-based image dataset, SimgFall, from the existing accelerometer or gyroscope-based sensor data of the SiSFall dataset for the early detection of falls to accelerate the medical assistance process. The SimgFall dataset was used to train and evaluate the FallCNN model, a novel deep Convolutional Neural Network (CNN) architecture comprising multiple CNN folds to effectively learn discriminative features from the transformed signal representations. These models utilize depth-wise convolution with varying dilation rates to efficiently extract diversified features from the SimgFall dataset. The dataset contained 1992 signal-based images, of which 498 were samples collected for fall, jump, stumble, and walk for the 4 classes. The initial architecture, referred to as FallCNN_1, with two basic convolutional layers and max-pooling, which is simple and efficient in feature extraction and dimensionality reduction, resulted in 94% accuracy for detecting the 4 classes. The incorporation of the average pooling and dropout layers in FallCNN_2 reduced overfitting and improved feature extraction, thereby enhancing the accuracy to 95%. Expanding the feature dimensions in FallCNN_3 further refined the capacity of the model to capture intricate patterns, achieving a notable accuracy of 97%. Finally, FallCNN_4 with three convolutional blocks and additional intermediate layers achieved the highest accuracy of 98%, demonstrating cumulative performance improvements through architectural enhancements. Furthermore, the performance of the generated dataset using different pretrained and custom models was evaluated based on the loss and accuracy curves. The experimental results showed that the highest classification accuracy was 98%, with a loss of 0.0833, using categorical cross-entropy as the loss function.

## 1 Introduction

In today’s world, falls are one of the most common causes of death, particularly among older adults, due to their physical weakness. Research by the World Health Organization (WHO) suggests that 42% of people above the age of 65 years suffer from at least one fall (World Health Organization, 2007). Therefore, as age increases, older adults’ fall rates also increase. Although there are a lot of fall detection devices available in the market, the majority of them are expensive, non-portable, or inaccurate.

The fear of falling is more common among people who live alone because it takes a long time to obtain assistance in the event of a fall (Lord et al., 2010). Individuals who do not receive medical help within the first hour of an accident are more likely to die or develop chronic illness. In addition, 50% of older adults who lay on the floor for a long time after a fall without medical assistance died within 6 months of the date of the accident. To overcome this and assist older adults, a more efficient fall detection system must be developed. This system should be inexpensive, portable, and accurate, and should provide timely alerts to minimize the effects of delayed medical care and reduce the fear of falling (Igual et al., 2013).

**Figure 1:**
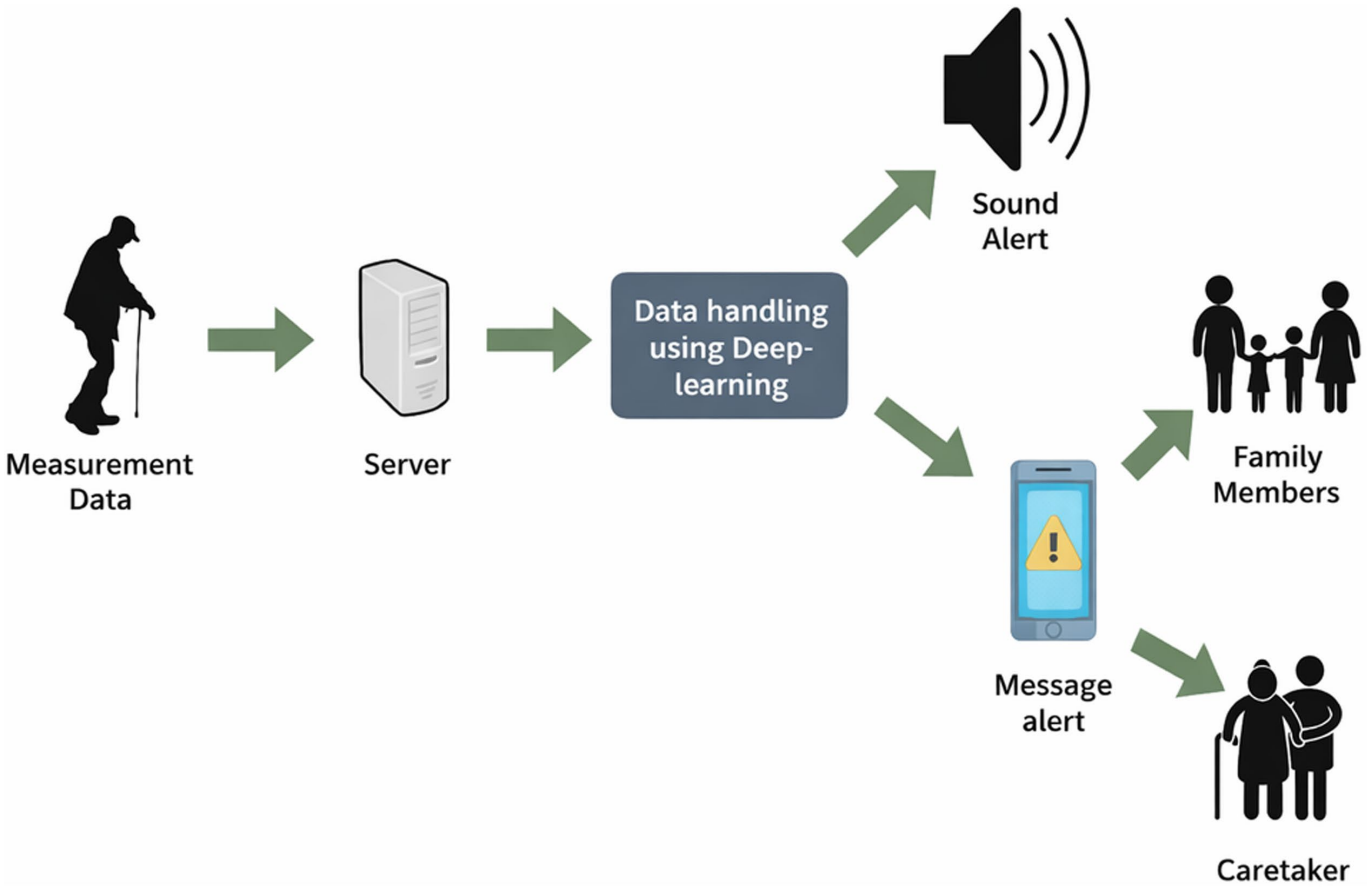
Fall detection among elderly persons (FDEP) model.

Figure 1 shows the Fall Detection among Elderly Persons (FDEP) system, which is generally followed to alert caretakers and family members if any fall occurs. The available fall detection systems are classified into various categories depending on the devices and intelligent algorithms involved: wearable sensor-based methods, non-wearable sensor-based methods, and Artificial Intelligence (AI)-based image or video processing methods, such as machine learning and deep learning models. Non-wearable-based sensors are invasive and do not solve the issue of adults living alone. Non-wearable vision-based devices, such as cameras, suffer from privacy concerns, high installation costs, and a lack of portability, despite achieving reasonable accuracy and reliability. These systems must be physically installed at specific locations to limit their effectiveness when the user is away from home. Furthermore, nearly half of all falls among older adults occur outside the home, making wearable sensor-based fall detection systems combined with intelligent AI learning models suitable for continuous real-world fall monitoring (Mubashir et al., 2013).

Convolutional neural networks (CNNs) have achieved great success in fields such as object recognition and image classification with large training datasets. Hence, deep learning could be applied for classifying movements of older adults with sufficient data extracted from accelerometer and gyroscope (Montesinos et al., 2018) end devices. This study generated a signal-based image dataset, SimgFall, from the existing accelerometer or gyroscope-based sensor data of the SiSFall dataset for the early detection of falls to accelerate the medical assistance process. A deep learning-based solution for fall detection is proposed for the generated SimgFall signal images and is trained using custom CNN and transfer learning models, and the performance is analyzed.

The proposed FallCNN model addresses the following research problems.

1. The majority of existing studies explore hardware-based wearable or non-wearable sensor data rather than an AI-based architecture for fall detection.
2. Performance improvements should be achieved by enhancing existing IoT-based models.
3. Limitations in the affordability of the hardware models for fall detection have to be addressed.
4. Due to the lack of a fall dataset, it is very difficult to develop a deep learning solution for the fall detection problems of older adults.

The main objective of this study is to propose a replacement strategy in which deep learning models can be trained using graph images generated from the SiSFall dataset’s sensor data, named SimgFall. Separate SimgFall images are created for each activity, which are then used to train and test the model (FallCNN and transfer learning models). The proposed system examines four areas of behavior: falling, jumping, walking, and stumbling. The major contribution of the proposed FallCNN system is

- A replacement strategy for the hardware model adopted by the majority populations was proposed and successfully devised using a deep learning model.
- The proposed unique custom CNN models, such as FallCNN_1, FallCNN_2, FallCNN_3, and FallCNN_4 deep learning architectures, were successfully implemented for the generated SimgFall dataset.
- The proposed model is compared with the two transfer learning models MobileNetV2 0.05 and MobileNetV2 0.1, all of which are trained and tested with the SimgFall dataset.
- To assist in better learning for extracting elite patterns to be categorized, the FallCNN model includes multiple convolution models with effective activation layers.
- The results for both custom FallCNN and pretrained models with the SimgFall dataset are shown in the form of ROC curves, accuracy, and loss for both training and validation, respectively.

The remainder of this study is organized as follows: Section 2 summarizes previous studies in the field of fall detection, whereas Section 3 details the proposed studies and architectures. The experimental analysis and results of the custom FallCNN and transfer-learning models are presented in Section 4. Section 5 concludes the study with a future scope.

## 2 Literature review

Pannurat et al. (2014) analyzed fall detection based on wearable sensor-based methods and ambient sensor-based methods. Sensors such as gyroscopes, pressure sensors, accelerometers, and microphones are used to detect early falls. Accelerometers are extensively used.

A technique based on near-field floor imaging sensors was proposed by Rimminen et al. (2010), where a two-state Markov chain model was used to classify activities, and pose estimation was performed using Bayesian filtering. Gjoreski et al. (2011) proposed a method that uses an accelerometer sensor placed on the chest, thigh, ankle, and waist to detect falls. The results showed that the sensors placed on the chest or waist were effective in detecting falls, whereas the combined results achieved the best results. Li and Stankovic (2011) detected falls by collecting the information from sensors mounted on furniture (bed and chair) along with the data obtained from the accelerometer placed on the person’s thigh, wrist, ankle, chest, and forearm.

The most commonly used ambient sensors are piezoelectric sensors, acoustic sensors, infrared sensors, cameras, and Kinect RGBD cameras (Stone and Skubic, 2015). Tzeng et al. (2010) used an infrared camera to distinguish the subject’s behavior and a pressure sensor to detect the pressure on the floor. Cameras are widely used among ambient sensors, and the rapid growth of computer vision has boosted the use of video-based fall detection methods (Mubashir et al., 2013). Challenges faced during the detection of falls in the small available population (older adults), regardless of the acquisition strategy, decrease their accuracy in real-world applications. Very few publicly accessible datasets that include data on falls and activities of daily living (ADL) are available for research. Several studies (Vavoulas et al., 2014; Medrano et al., 2014) have analyzed and detected the motion of a person for the prediction of falls. Since the self-developed embedded system can be easily replicated, SisFall data (Sucerquia et al., 2017) are considered the most accurate data for fall and ADL analysis among the available data.

Computer-assisted algorithms to detect falls have been widely researched using rule-based methods, machine learning, and artificial intelligence-based models. The majority of the models analyzed data obtained from wearable sensors using a thresholding technique or a set of rule-based predictions (Kau and Chen, 2015). Sannino et al. (2015) devised a rule-based supervised method to analyze the accelerometer data, which decided whether the given event was a fall or not. Logistic regression (LR), support vector machine (SVM), neural networks (NNs), multilayer perceptrons (MLPs), regularized discriminant classifier (Howcroft et al., 2018; Rodrigues et al., 2018), nearest neighbors (KNNs), naive Bayes classifier (NB), and random forest (RF) (Palmerini et al., 2020) are used for estimating fall risk, where these approaches use notable individual characteristics to assess the fall.

In addition, inertial sensors, which use sophisticated machine learning approaches, are used to determine fall risk, resulting in low classification accuracy (Silva et al., 2017). If the system classifies a patient with a high risk of falling as low risk, there is a possibility of severe consequences, deterring much-needed treatment. As a result, achieving high accuracy is difficult, necessitating the use of powerful deep learning techniques. Nunez-Marcos et al. (2017) presented a 2D CNN model using an optical image, attempting to integrate motion information via optical flow for efficient fall detection. Deep learning techniques, such as convolutional neural networks (CNN) (LeCun et al., 2015), have gained huge success within the last few years in fields such as natural language processing (NLP) and object detection. Deep learning, on the other hand, can learn an efficient representation (function) automatically, which prompted the use of a CNN to perform an efficient fall detection technique in this study. Lu et al. (2018) analyzed 3D CNN for the fall and no-fall data, achieving higher accuracy.

Adhikari et al. (2017) examined the body positions of individuals lying down after falling on a sofa or crawling, using a Convolutional Neural Network (CNN). The researchers examined various image combinations, including RGB, depth, RGB-D, and background-subtracted RGB-D, using their dataset. An optimal approach for monitoring indoor video-based falls was accomplished by integrating background-subtracted RGB and depth pictures with a Convolutional Neural Network (CNN). Santos et al. (2019) introduced a Convolutional Neural Network (CNN) to examine three publicly available datasets (URFD, Smartwatch, and Notch). They assessed various performance measures, such as accuracy, precision, sensitivity, specificity, and the Matthews Correlation Coefficient. Their study attained an accuracy of 85.71% for the URFD dataset, 98.43% for the Smartwatch dataset, and 86.76% for the Notch dataset. Espinosa et al. (2019) examined the identification of falls by utilizing a 3D Convolutional Neural Network (CNN) and an optical flow technique to gather data on the relative movement between consecutive photos. They achieved a high accuracy of 95.64% on the publicly available UP-Fall identification dataset.

Wu et al. (2021) introduced a CNN-Causal LSTM network comprising an encoding layer, a decoding layer, and a ResNet18 classifier. This network attained a remarkable accuracy of 99.79% when evaluated using the SisFall dataset. Sadreazami et al. (2021) conducted time-frequency analysis by utilizing the short-time Fourier transform to produce spectrograms. The spectrograms were transformed into binary images and examined using a Convolutional Neural Network (CNN) to distinguish between fall and non-fall activities. This method achieved a specificity of 97.82%. Alanazi and Muhammad (2022) introduced a streamlined and efficient CNN model for detecting human falls. They utilized 6,392 sequences generated from the Le2i fall detection dataset. The model attained an accuracy of 99.03%, a sensitivity of 99.00%, a specificity of 99.68%, and a precision of 99.00%. Galvão et al. (2021) examined a deep learning model for the UP-Fall dataset, utilizing multimodal CNN data to decrease the occurrence of false positive events. The model achieved an accuracy of 99.87%. Alanazi et al. (2023) employed a refined human segmentation model and an image fusion technique to preprocess and categorize a collection of live footage using a 3D multi-stream CNN model (4S-3DCNN) on the Le2i dataset. Their results showed an accuracy rate of 99.44%, a sensitivity rate of 99.12%, a specificity rate of 99.12%, and a precision rate of 99.59%. These findings contribute to the prevention of problems related to fall injuries. Galvão et al. (2021) introduced a thorough model for Human Activity Recognition (HAR) and fall detection called PCNN-Transformer. This model uses smartphone sensors as the basis. The PCNN-transformer uses a parallel architecture that combines CNN blocks and transformer encoders. It incorporates a residual mapping method to extract and emphasize relevant characteristics. A comprehensive assessment was carried out using three publicly available Human Activity Recognition (HAR) datasets: SisFall, UniMib-SHAR, and MobiAct. The model achieved accuracies of 99.95, 98.68, and 99.71% in the given order.

Durga Bhavani and Ferni Ukrit (2024) introduced the INDCNN-FDC model, which utilizes deep transfer learning with the Inception v3 model to classify events into two categories: fall and non-fall. The model achieved an accuracy of 97.66% on the UR Fall Detection dataset. Ha et al. (2024) introduced a fall detection framework that combines a Mixture of Experts (MoE) and CNN3D models on the UP-Fall Detection dataset. This approach achieved an impressive weighted average F1-score of 99.67%. Kandukuru et al. (2024) employed a Convolutional Neural Network (CNN) to analyze optimized optical flow pictures. These images were preprocessed in both the spatial and frequency domains to capture different body positions, such as standing, sitting, walking, and falling. The results were highly accurate and precise.

Gao et al. (2024) and Gao et al. (2025) explore various techniques for enhancing 3D object detection in autonomous vehicles, including monocular depth perception, pseudo-LiDAR data conversion, and channel attention mechanisms. Gao et al. (2023a) and Gao et al. (2023b) proposed a method for anomaly detection in IoT time-series data using a memory-augmented autoencoder.

Patel et al. (2026) proposed a privacy-preserving fall detection system using ultrasonic sensors to address the limitations of cameras and wearable devices. The hybrid deep learning model combining RNN, LSTM, and Bi-LSTM achieved 98.14% accuracy on ultrasonic time-series data. The system was further integrated with the SAGIN framework to obtain reliable connectivity, low latency, and real-time fall detection in remote areas. Bach et al. (2026) introduced FallNet, a lightweight fall detection model for real-time edge deployment. The system enhances the YOLOv8n pose with DyC2F blocks and an integrated LSTM for early fall prediction using keypoint trajectories. The model obtained 92% precision, 97% recall, and a 94% F1-score in the COCO dataset. Sykes (2025) developed a fall detection system using human pose estimation and transformer models designed to operate on low-power devices while preserving user privacy, attaining 98% accuracy with high sensitivity and precision.

Kim et al. (2025) developed a construction site fall detection system using a skeleton AI model called YOSAP-LSTM. The method combined YOLOv8 for human detection, SORT, and AlphaPose for keypoint tracking and a one-dimensional (1D) CNN-LSTM classifier, attaining 98.66% accuracy. Jarrah et al. (2025) developed a sensitive ADL and fall detection dataset focused on older adult individuals using low-cost wearable IMU sensors placed on the waist and thigh. A CNN-based model achieved high performance with an average accuracy of 98.97% due to synchronized dual-IMU data. The model was successfully deployed on a wearable embedded system, thereby demonstrating reliable real-world fall detection. Sumathi et al. (2025) proposed an IoT-enabled wheelchair fall detection system enhanced with blockchain for secure logging of fall events, including time and location. Yu et al. (2025) introduced TCNTE, a real-time Temporal Convolutional Network (TCN) with a Transformer Encoder (TE) to improve robustness while remaining lightweight for edge deployment. The model achieved high accuracy, with UP-Fall up to 99.58%, Le2i 97.01%, and GMDCSA-24 92.99%. Integrated with YOLOv8 pose estimation and BoT-SORT tracking, TCNTE runs efficiently on an NVIDIA Jetson Orin NX at 17–19 fps, demonstrating strong real-time performance for practical care applications in older adults.

## 3 Proposed study

**Figure 2:**
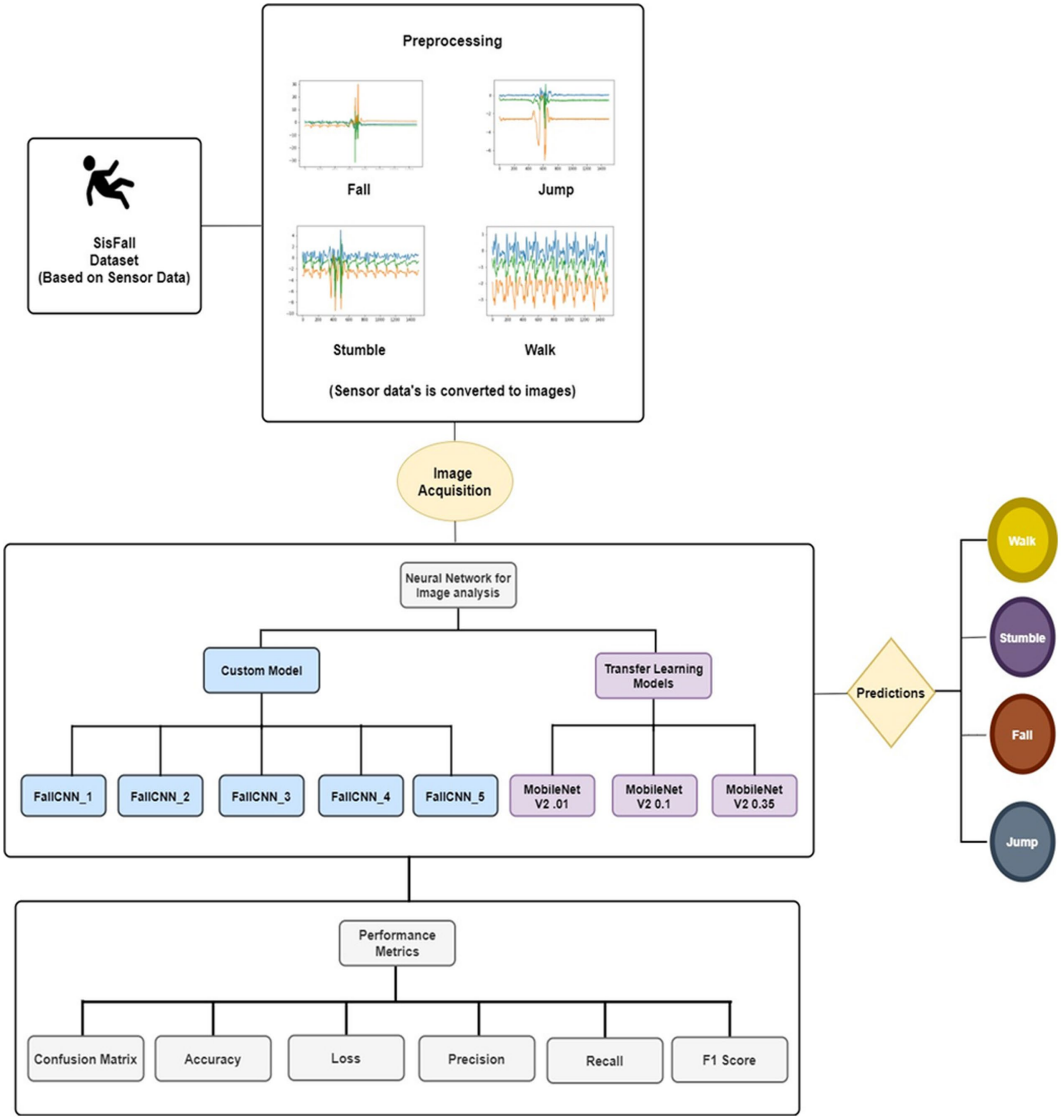
The architecture of fall detection of elderly persons (FDEP).

In this study, custom CNN models in conjunction with transfer learning models were analyzed to detect the movement of older adults. This study proposes a unique approach in which models are trained using signal images plotted from the sensor data of the SiSFall dataset named SimgFall. For each older adult’s movements, such as falling, jumping, walking, and stumbling, separate SimgFall images were generated and used to train and test the custom model. The proposed system architecture for fall detection among older adults is shown in Figure 2.

### 3.1 Dataset

SisFall dataset (Sucerquia et al., 2017) includes sensor data detected using sensors such as ADXL345, ITG3200, and MMA8451Q, where ADXL345 and MMA8451Q are accelerometer sensors, whereas ITG3200 is a gyro sensor. The dataset was generated with the assistance of 38 volunteers divided into 2 groups: older adults and young people. Young adults perform both falls and other activities, whereas some older adults do not perform some activities because of health issues. Multiple trials of the same activity, such as older adults’ movements, including falling, jumping, walking, and stumbling, were performed by the selected individuals and were available in the SisFall dataset.

### 3.2 Generation of SimgFall dataset

**Figure 3:**
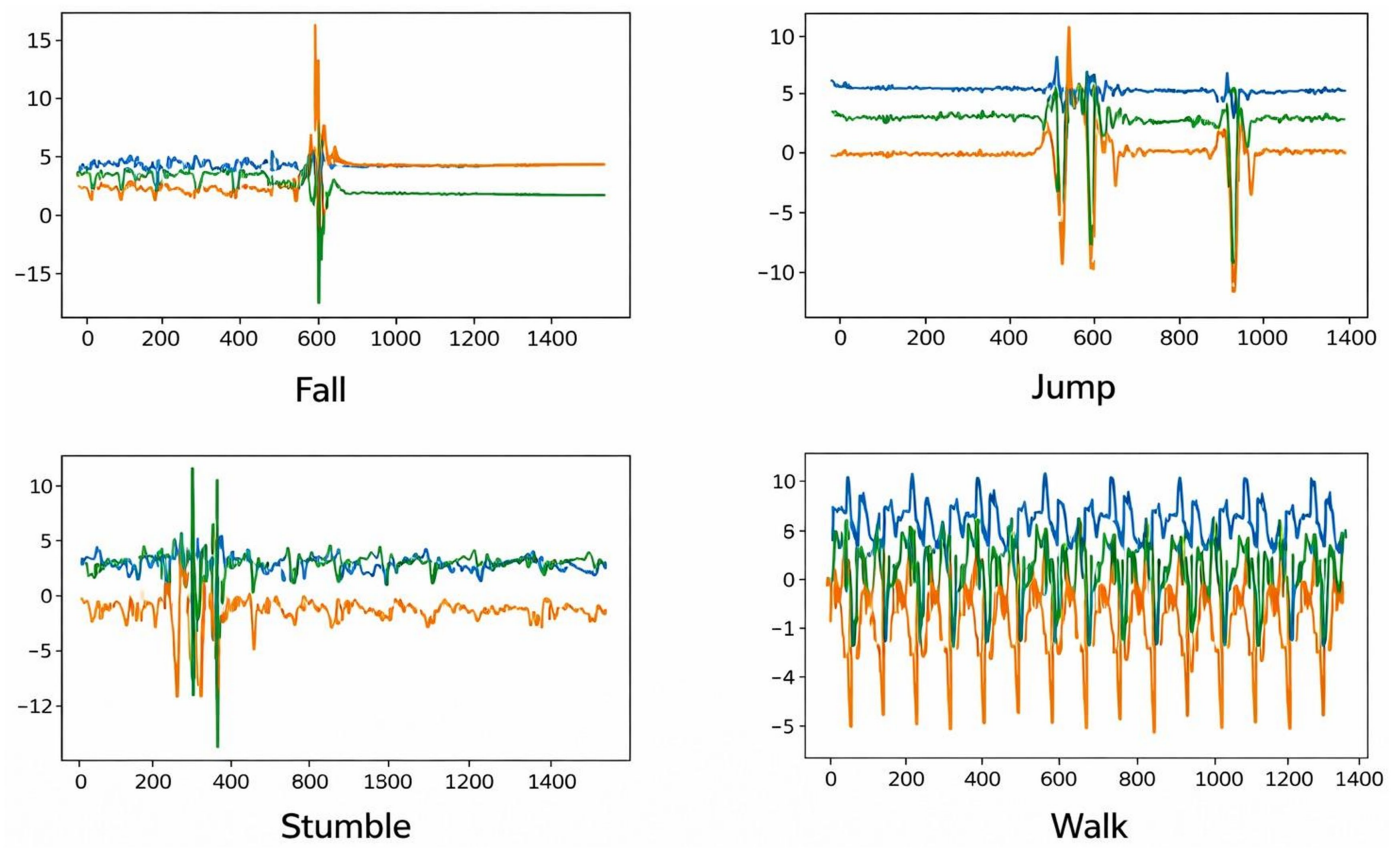
Sample SimgFall images created from plotted IMU accelerometer and gyroscope signals.

Data preprocessing is an important process that must be performed in the majority of data science-related studies. Preprocessing is commonly used to eliminate null values and unwanted rows or columns from a dataset. From the SisFall dataset, we only considered 1992 CSV files because some volunteers had not performed all the activities required; only the CSV files of individuals who performed all the necessary activities were considered for the experiment. From the selected CSV files, the first three columns of the CSV file that correspond to accelerometer sensor data are selected, and all the remaining columns are eliminated. The first three columns of each CSV file are plotted and saved as separate signal images in their corresponding classes: fall, walk, jump, and stumble. A total of 1992 signal images were generated from 1992 CSV files of the SisFall dataset, named SimgFall. The SimgFall dataset is then divided into training and validation data and fed to the CNN models. In the proposed study, sensor-based SisFall data is converted to images to explore fall detection from a new perspective to improve performance. The generated images are categorized into four categories: fall, walk, jump, and stumble, where each image is scaled to 256 × 256. A total of 1992 images were captured from the SisFall dataset, which includes 498 images per category. The dataset creation procedure is given in Algorithm 1, and a sample of the generated SimgFall images denoting each class is shown in Figure 3.

Transforming sensor data into images offers several advantages over using 1D data. This allows the use of 2D convolutional layers, which can capture spatial relationships and complex patterns more effectively. Images also provide more intuitive and interpretable representations, making it easier to understand and visualize the data and decision-making process of the model. Additionally, pretrained CNN models on large image datasets can be fine-tuned on new image data, potentially enhancing the performance and reducing the training time. This conversion enables the application of more sophisticated neural network architectures, which can lead to improved accuracy and generalization. Furthermore, the images offer a consistent input shape and simplify the design and implementation of the neural network architecture. Collectively, these advantages contribute to the development of more effective and efficient fall detection systems.

**ALGORITHM 1:**
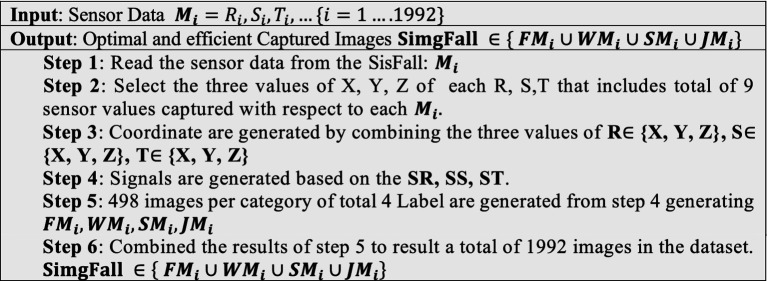
SimgFall dataset creation.

### 3.3 Convolutional neural networks (CNNs)

A convolutional neural network (CNN) consists of many neurones that work similarly to how neurones in the physical body work. A convolutional neural network includes an input layer, an output layer, and several hidden layers. In general, neurones in the CNN model are fed with input, followed by weighted sum computation, and forwarded to an activation function, resulting in the output of the model. Each image is initially considered as an array. In the proposed scheme, the color image is considered as a 3D array that consists of red, blue, and green colors, which have their own level of intensity. A convolutional function is applied to the image data to generate a feature map using a failure detector, and the convolutional operation is shown below.


$$F_{\mathrm{cov}}(x,y)=(D*F)(x,y)=\sum_{i}\sum_{j}D(x+i,y+j)F(i,j)$$

where D is the input matrix representing the input image, F is the filter of size x and y, and $\;F_{\mathrm{cov}}$ represents the output. In real life, humans can identify an object based on several features but might not look for all the features to identify an object. Similarly, in the case of a CNN, the object detector only considers the necessary features. After the feature map is obtained, a rectifier function is applied on top. The rectifier function Rectified Linear Unit (ReLU) is used because it increases non-linearity. The rectifier acts as a filter or function that breaks up linearity. The ReLU generates an output of zero for values less than zero; otherwise, it generates a raw output. A mathematical illustration of the ReLU function is given in the equation below.


$$R_{\mathrm{cov}}(x)=\max(0,x)$$

After this step, a pooling technique such as max-pooling/sum-pooling/mean-pooling is applied on top of it to generate a pooled feature map so that the model can easily identify the position of the object in the image. The size and number of parameters are also reduced in this step, thus preventing the model from overfitting. In general, the next step is flattening, in which the pooled features are converted into columns sequentially, one after another, so that they can be fed to a neural network for further processing.

**Figure 4:**
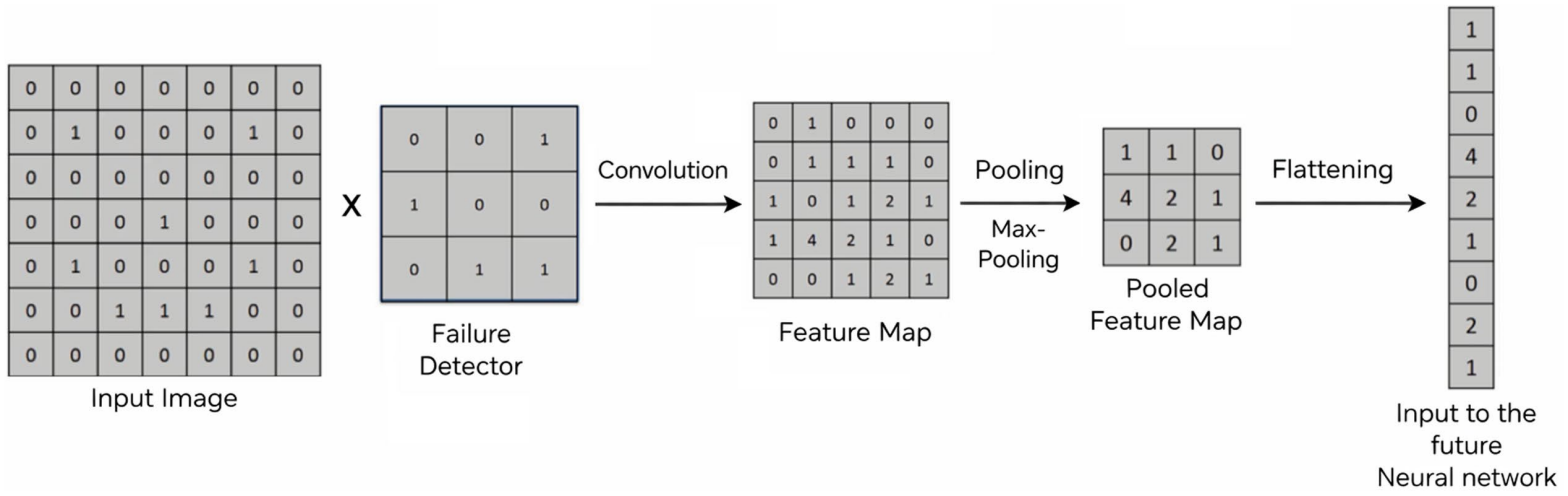
CNN model working example.

More layers can be added and can be connected to a dense layer that includes ‘n’ output nodes, depending on the number of classes. Each node in the final dense layer denotes the classes in the given input dataset. An example of the aforementioned operation is shown in Figure 4.

#### 3.3.1 Custom model

##### 3.3.1.1 FallCNN_1 model

**Figure 5:**
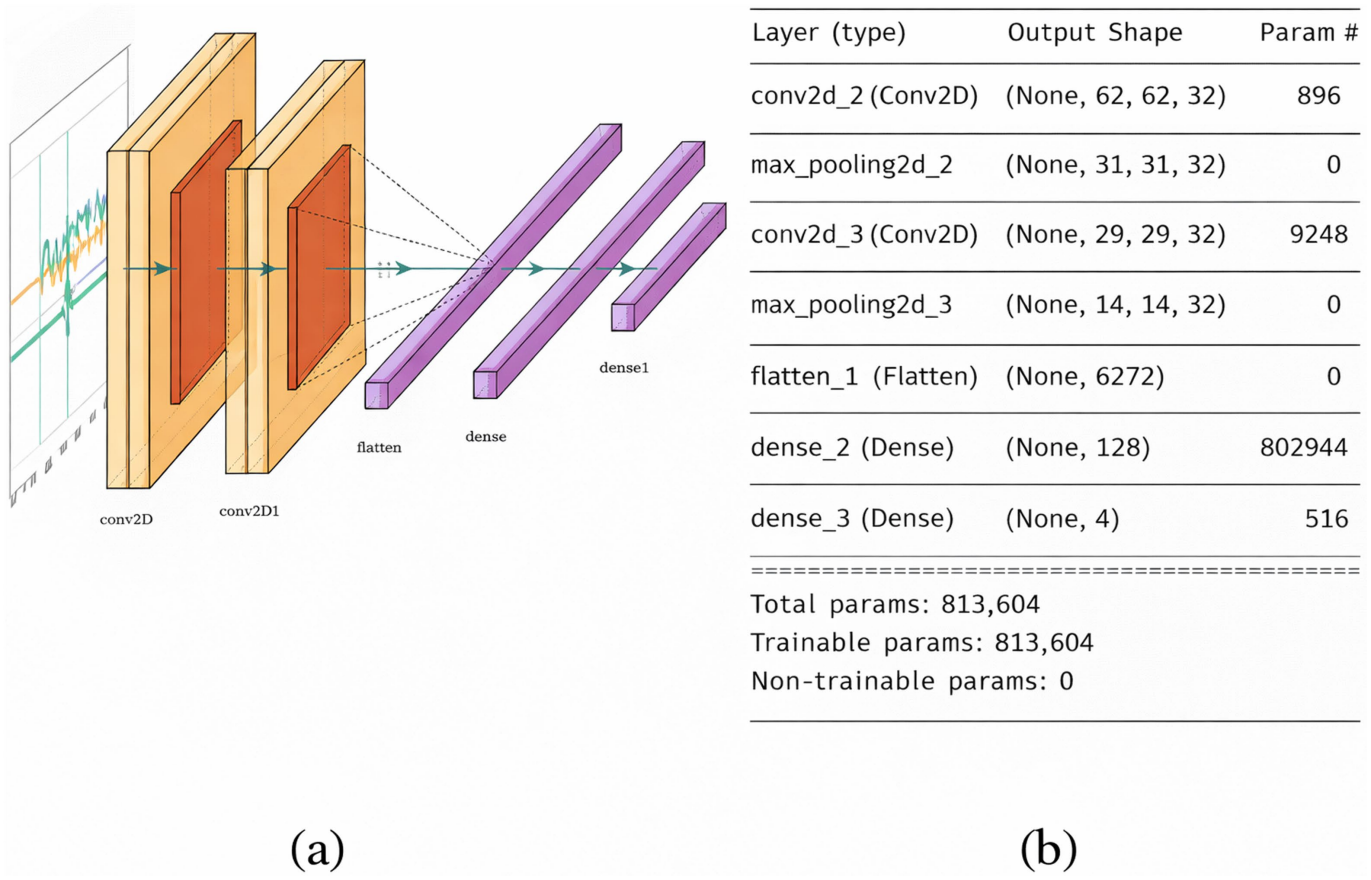
**(a)** FallCNN_1 model and **(b)** summary of FallCNN_1 model.

FallCNN_1 uses two 2-D convolutional layers and a max-pooling layer, in which ReLU is employed as an activation function. The primary CNN layer uses a 32, 3×3 essential filter processing the input data, resulting in a 32-feature map. The max-pooling layer uses a kernel size of 2×2, which results in dimension reduction. Likewise, to extract fine-scaled features of the image, a second convolution layer with a 32, 3×3 pixel filter was used, followed by a 2×2 kernel-based max-pooling layer generating 14×14×32 features. A flattening layer is employed to convert this output into a vector, which is further fed into a feed-forward neural network with 2 dense layers, with 128 hidden neurones and 4 output neurones. The activation functions that followed are ReLU for the hidden layer and sigmoid for the output layer. Categorical cross-entropy is employed as a loss function because the proposed analysis includes four categories. The FallCNN_1 model achieved a classification accuracy of 94% with a loss of 0.264 by training 813,604 parameters. Figure 5a shows the proposed flow diagram, and Figure 5b shows a summary of the FallCNN_1 model.

##### 3.3.1.2 FallCNN_2 model

**Figure 6:**
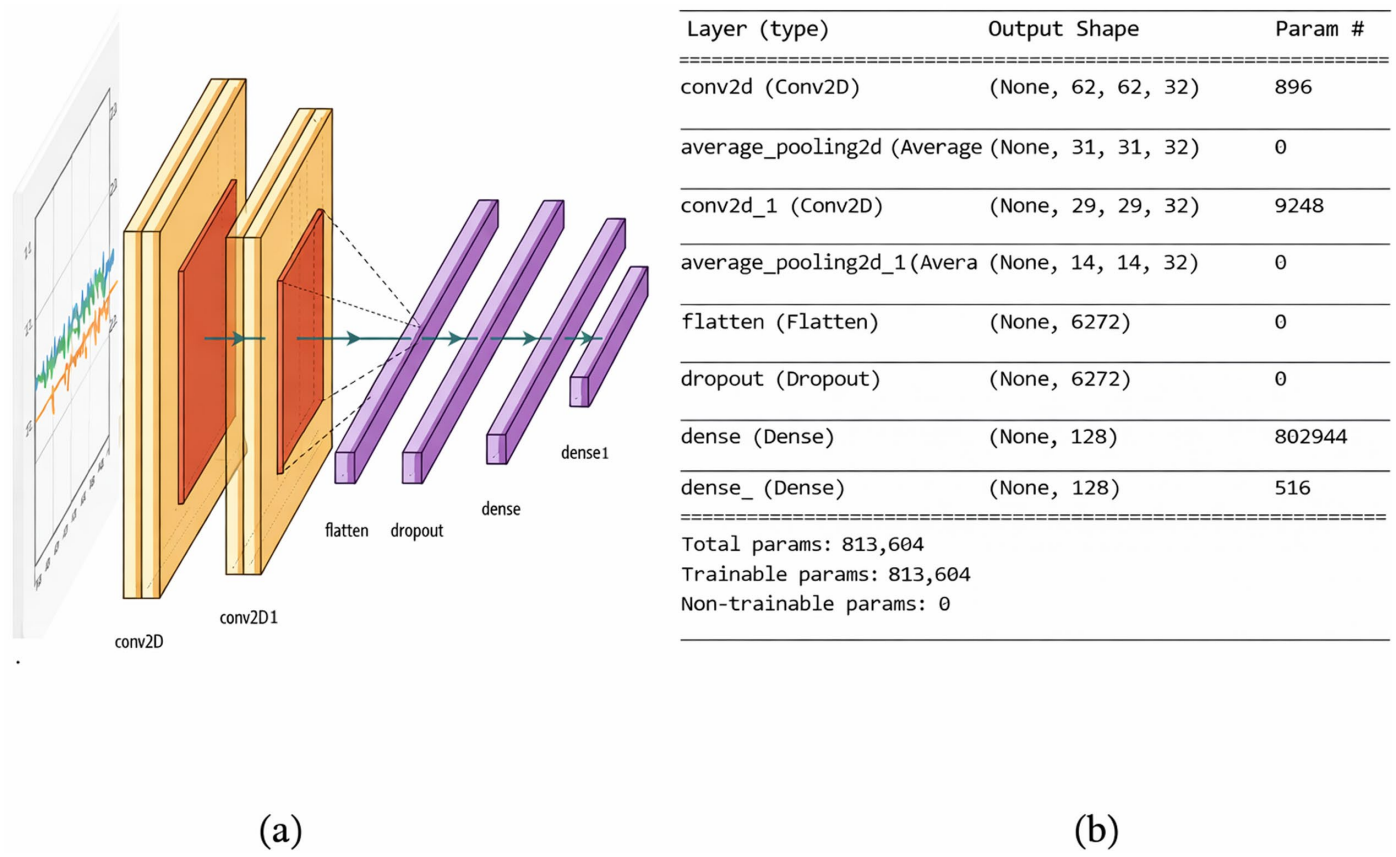
**(a)** FallCNN_2 model and **(b)** summary of FallCNN_2 model.

FallCNN_1 is modified with respect to the pooling layer, and a dropout layer is introduced, resulting in the FallCNN_2 model. FallCNN_2 uses the average-pooling technique, whereas FallCNN_1 uses the max-pooling technique. A dropout layer is used in this model to stop overfitting. A 20% drop allowed for the pruning of the less conducive neurones, which conjointly helps in reducing the generalization error. FallCNN_2 provided higher performance measures on the generated graph image dataset than FallCNN_1. The FallCNN_2 model achieved an accuracy of 95% with a loss of 0.118. Although the total parameters generated for this model and the previous one are the same, FallCNN_2 enhanced the accuracy by 1% due to average pooling and the dropout function. Figures 6a,b show the flow diagram and summary of the FallCNN_2 model, respectively.

##### 3.3.1.3 FallCNN_3 model

**Figure 7:**
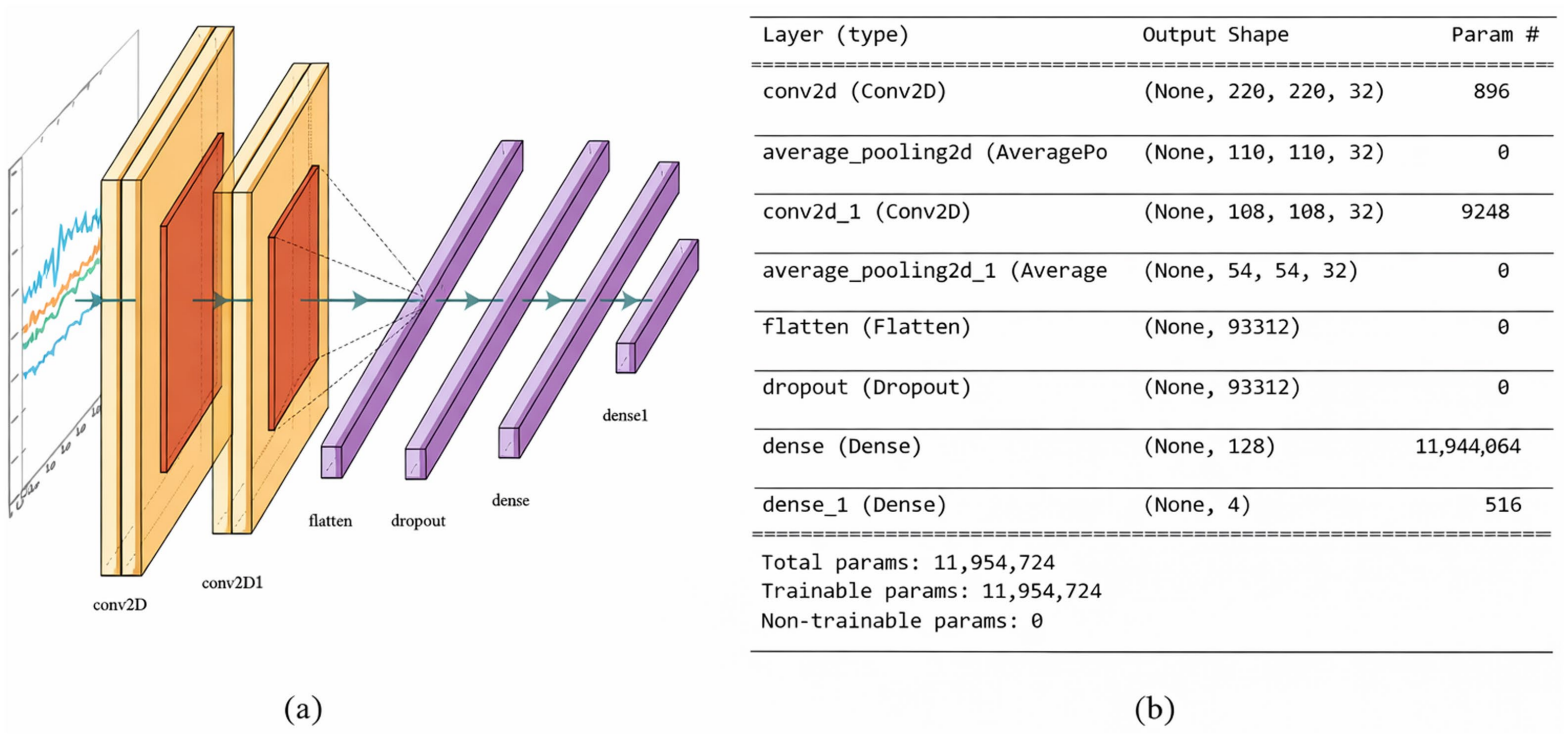
**(a)** FallCNN_3 model and **(b)** summary of FallCNN_3 model.

FallCNN_3 includes the same number of convolution, pooling, and dropout layers as the previous one, where the feature is remarkably increased from 62×62 to 220×220 in the convolution layers. The performance of the FallCNN_3 model achieved an improved accuracy of 97% with a loss of 0.098 by analyzing 11,954,724 features. The results show that Figures 7a,b show the flow diagram and model outline of the FallCNN_3 model, respectively.

##### 3.3.1.4 FallCNN_4 model

**Figure 8:**
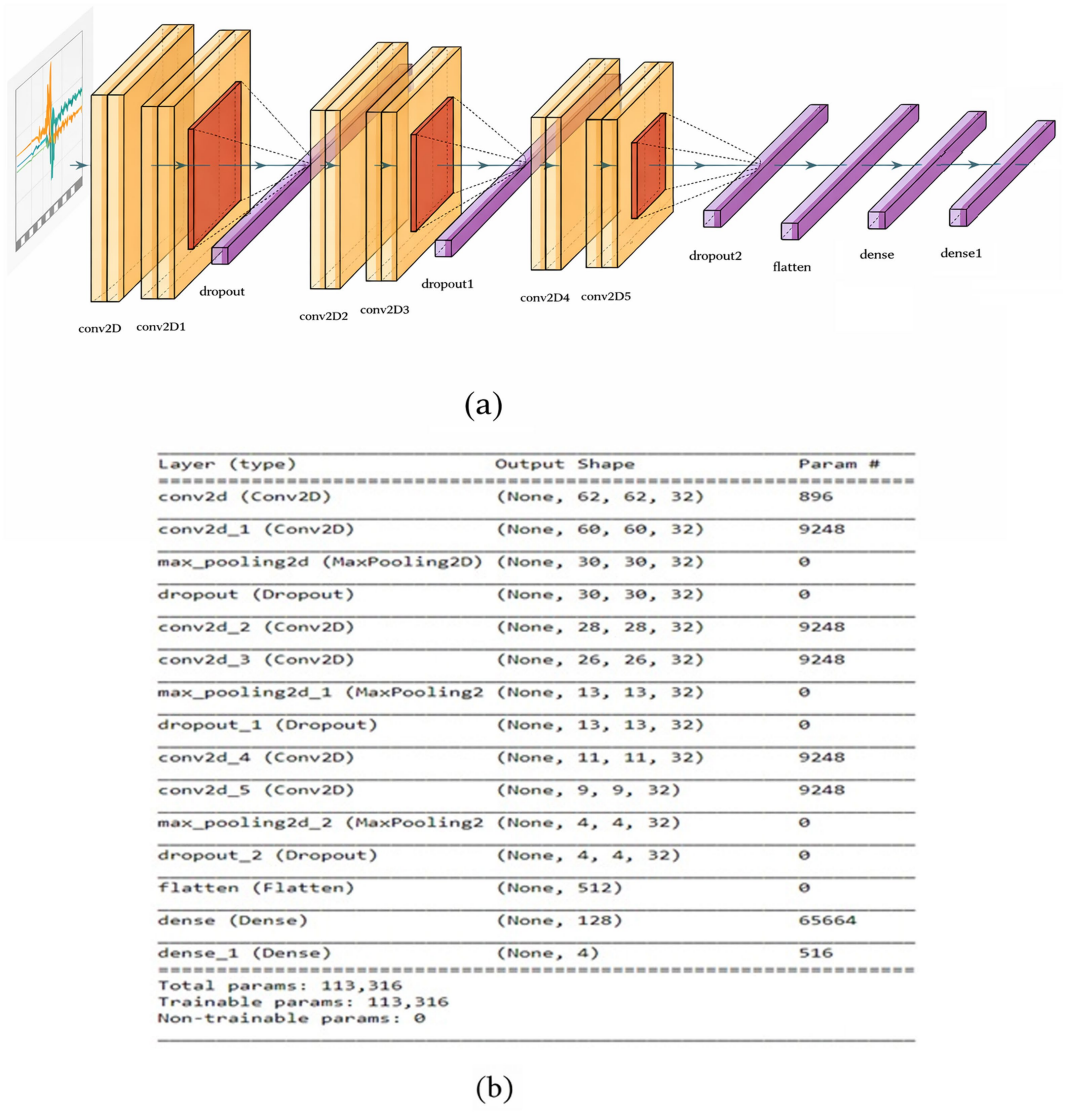
**(a)** FallCNN_4 model and **(b)** summary of FallCNN_4 model.

The FallCNN_4 model differs from the aforementioned models in terms of the number of layers and input parameters used. FallCNN_4 comprises three main convolutional blocks. Each convolution block is followed by another intermediate block. After each intermediate convolution block, a max-pooling/average pooling and dropout layer with a 20% drop rate is used to prevent the model from overfitting. The same structure is repeated three times, resulting in the highest accuracy of 98% with a loss of 0.0833. The FallCNN_4 model uses categorical cross-entropy as a loss function, similar to the other models. Figures 8a,b show the flow diagram of the FallCNN_4 model and its summary, respectively.

#### 3.3.2 Transfer learning models

The transfer learning model is used to apply the knowledge learnt from a task to solve another similar problem. In the proposed study, we created models with the help of the Edge Impulse platform, which is commonly used for machine learning on edge devices. Two transfer learning models, MobileNetV2 0.1 and MobileNetV2 0.05, are trained using the graphic images that are created with the help of the SiSFall dataset. MobileNetV2 is a lightweight convolutional neural network model that can classify images into 1,000 different categories. The two models, MobileNetV2 0.1 and MobileNetV2 0.05, considered for this research are designed in such a way that they have 10 neurones in their final layer with 0.1 dropouts. MobileNetV2 0.1 and MobileNetV2 0.05 achieved accuracies of 97.2 and 97.5%, respectively. MobileNetV2 0.05 has a slightly improved performance over the MobileNetV2 0.1 model. These transfer learning models have some problems distinguishing certain activities properly.

## 4 Results and discussion

This section presents a comprehensive overview of the dataset, experiments, model training, and validation. A performance comparison between the proposed approach and existing methods is also presented in this section.

### 4.1 Model training and validation

The proposed FallCNN and transfer learning models are trained using graph images generated from the SisFall dataset. The distribution of the samples into the training and validation datasets is in the ratio of 1,591:401, i.e., 1,591 images for training and 401 images are used for testing purposes. The target data with 1,591 images includes 1,378 samples of fall, 88 samples of jump, 89 samples of stumble, and 36 samples of walk class. Validation data with 401 images includes 345 samples of fall, 23 samples of the jump, 18 samples of stumble, and 10 samples of the walk class. The models were trained for 50 epochs. The model is evaluated on various classification metrics that include accuracy, confusion matrix, F1-score, recall, and precision. Table 1 shows the training and validation accuracy and loss of the custom FallCNN models. As the plots indicate the proper training design rather than only the final performance, the original axis scales are retained in the graphical results.

**Table 1:** Learning process recorded by the custom CNN (FallCNN) models, considering the number of epochs, model loss, and model accuracy curve.

Model	Training and validation loss	Training and validation accuracy
FallCNN_1	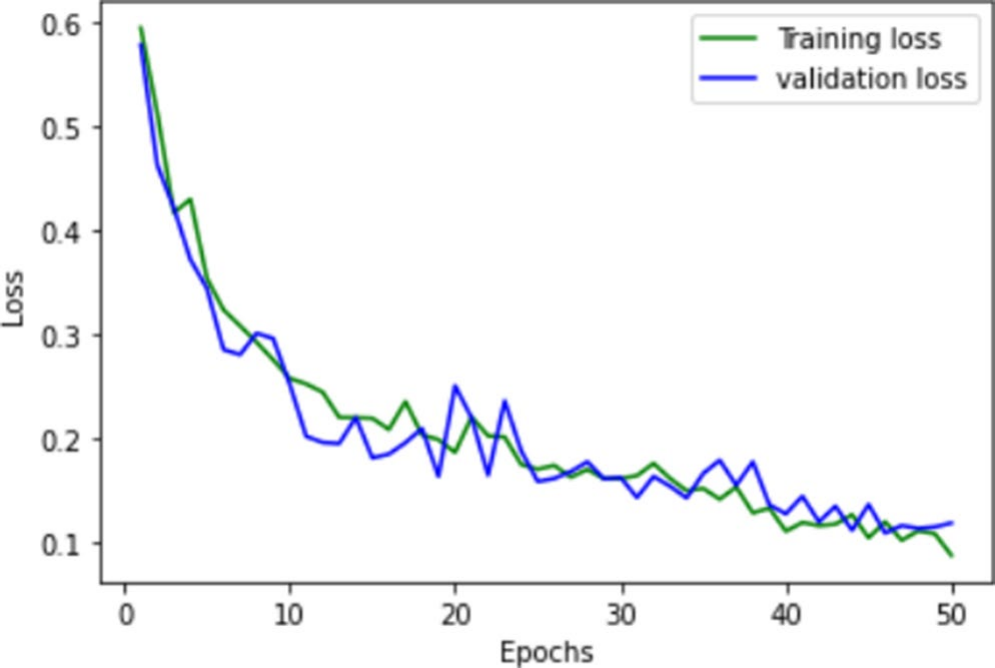	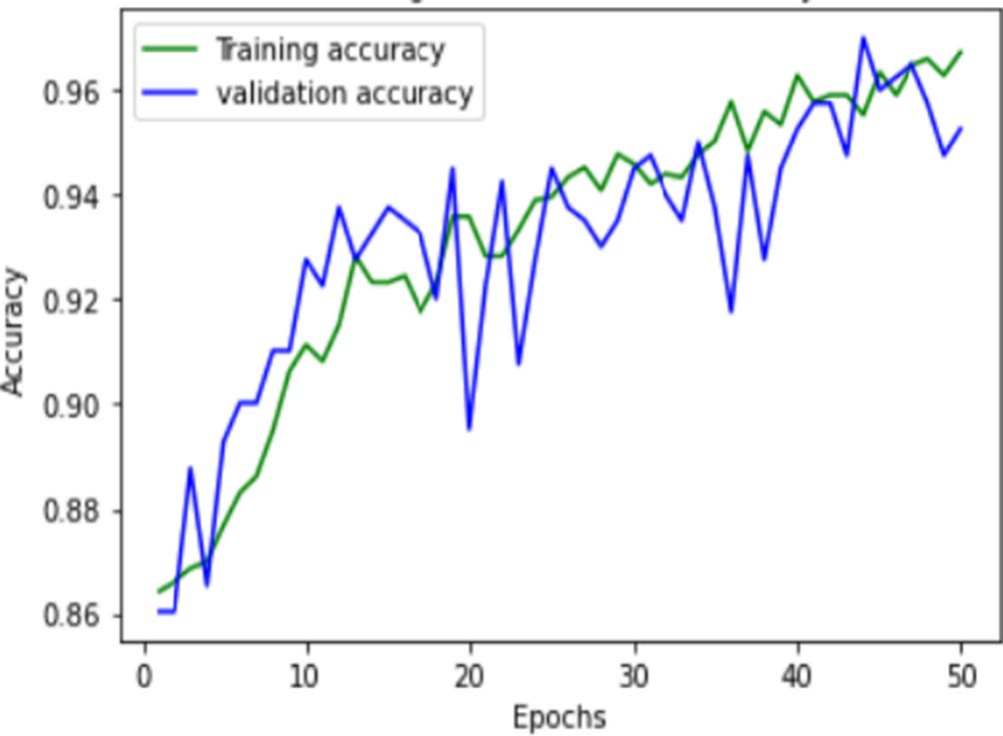
FallCNN_2	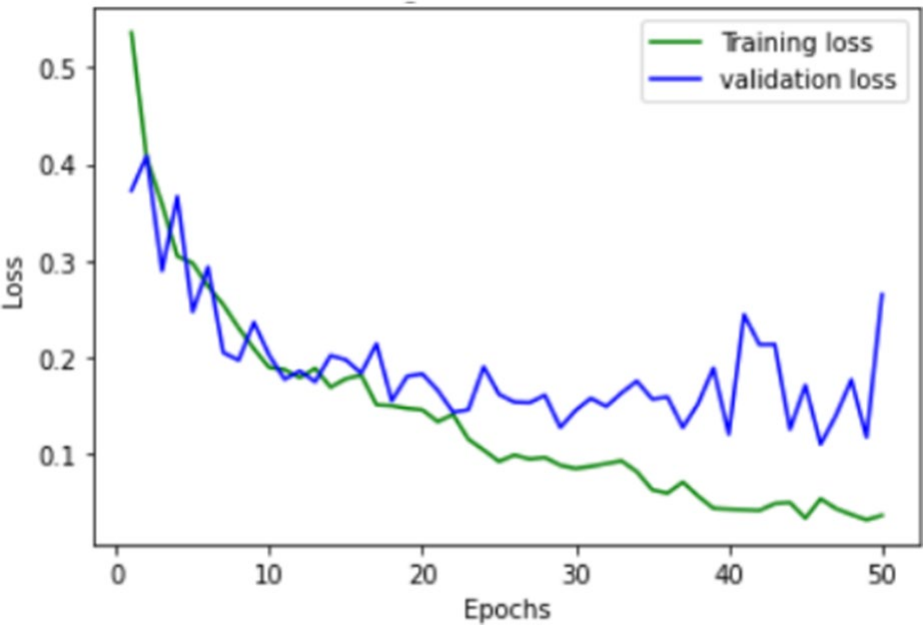	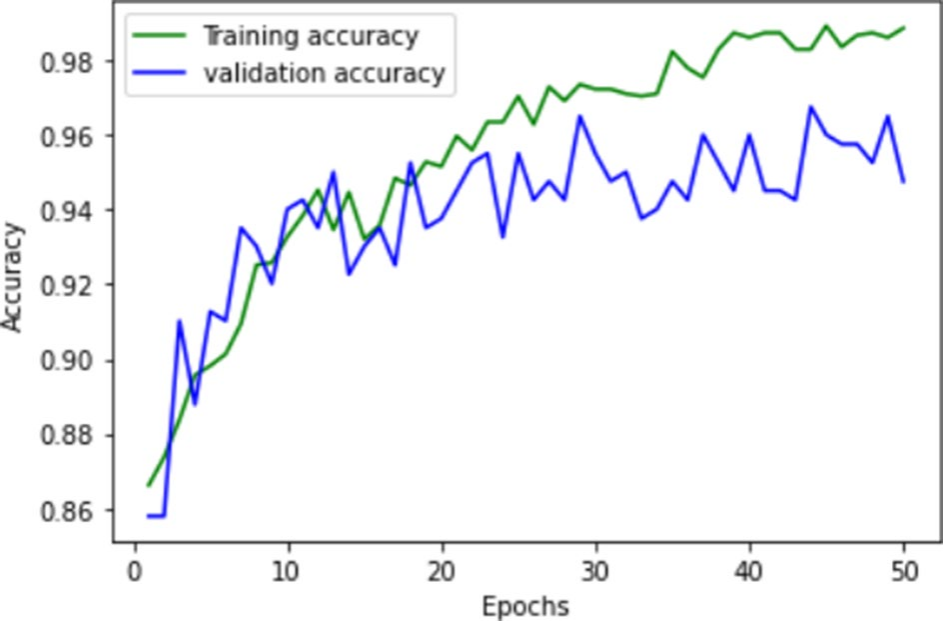
FallCNN_3	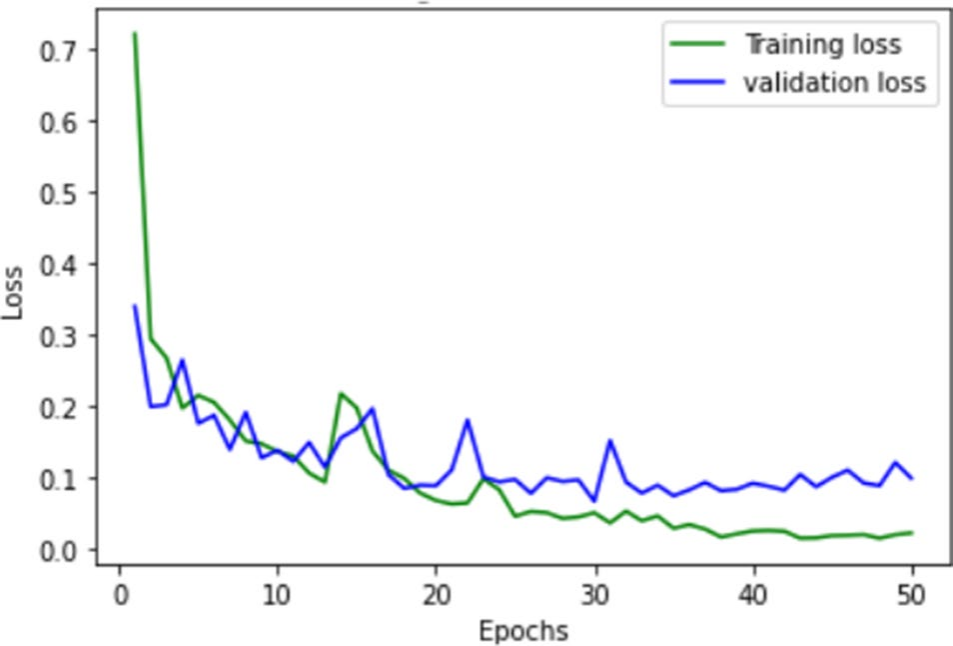	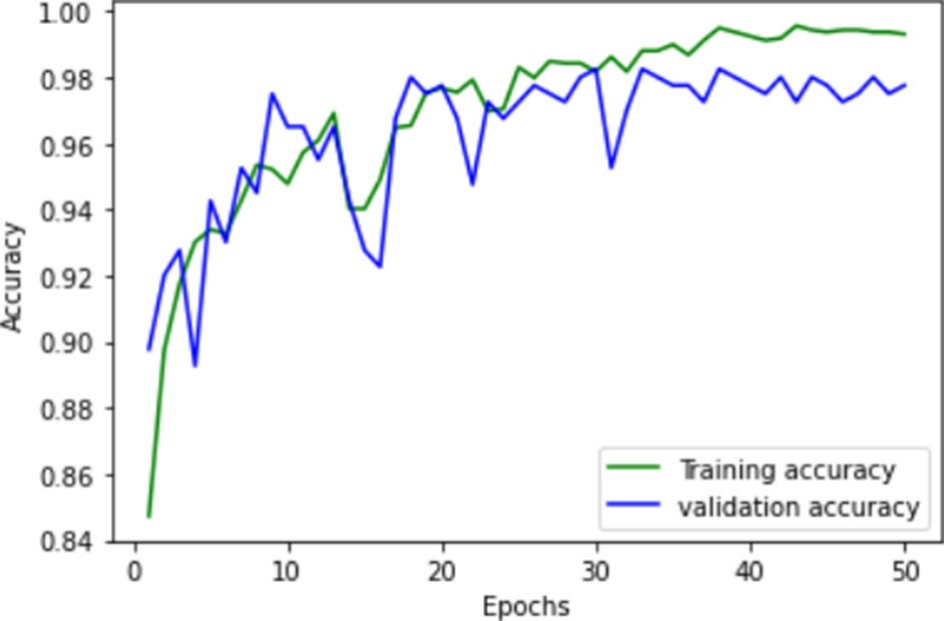
FallCNN_4	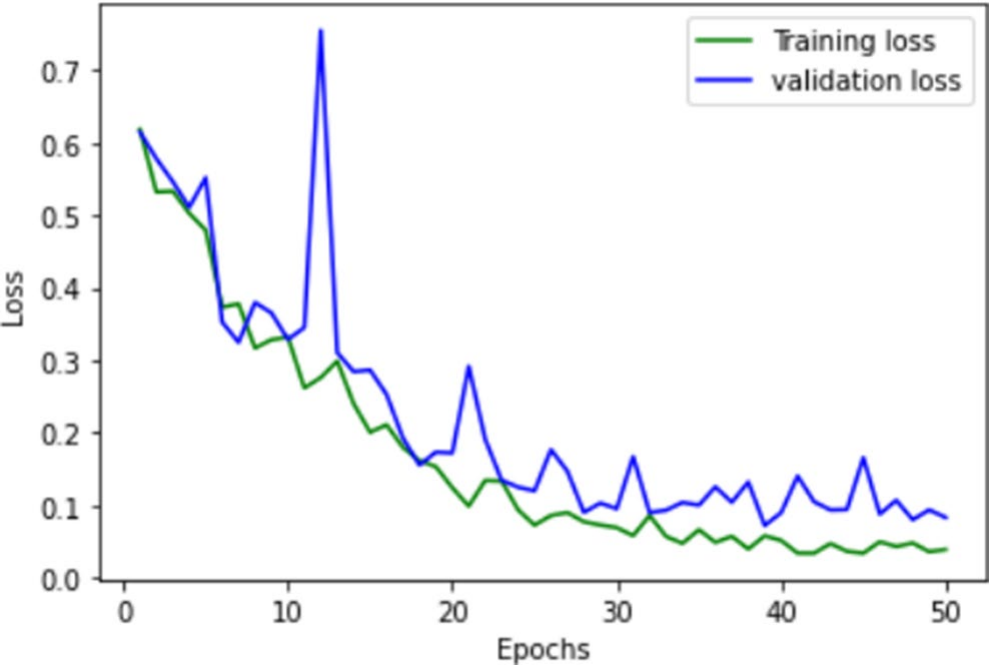	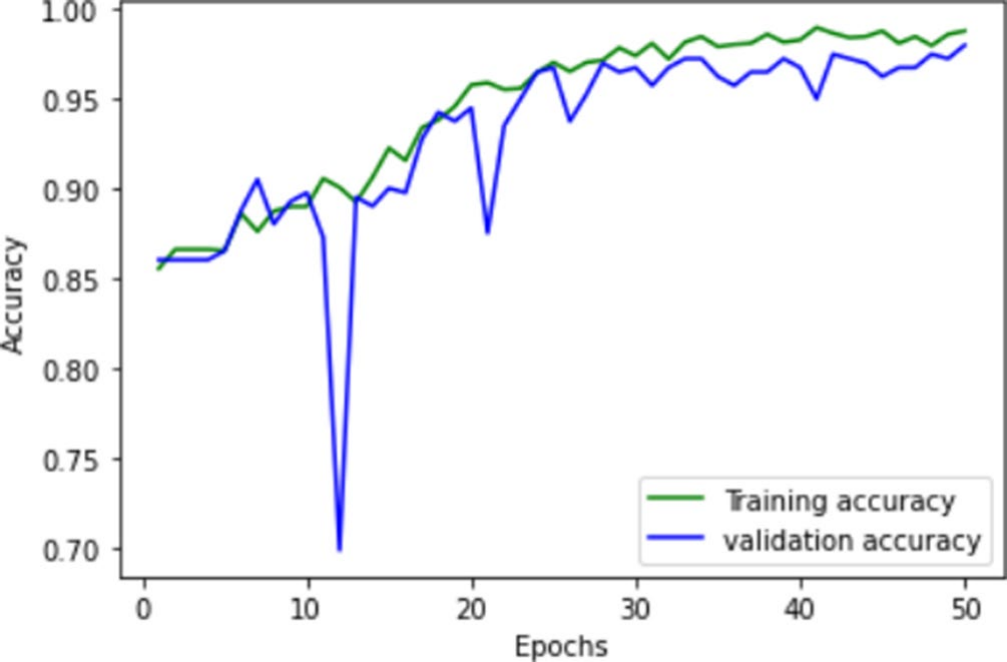

Optimal selection of the number of epochs is essential for training a deep learning model because both excessively low and excessively large epoch counts have disadvantages. An inadequate number of epochs can result in underfitting, limited ability to generalize, and insufficient acquisition of patterns, leading to subpar performance on both the training and validation datasets. Conversely, excessive epochs can lead to overfitting, a situation in which the model acquires knowledge of irrelevant patterns in the training data, thereby diminishing its capacity to make accurate predictions on fresh data. In addition, increasing the number of epochs results in extended training durations, which can be both computationally costly and time-consuming, particularly when dealing with extensive datasets and intricate models. Once a particular threshold is reached, additional epochs may result in negligible differences. Hence, achieving the optimal performance of the model requires a crucial process of identifying the appropriate equilibrium through validation and experimentation. We evaluated the model with epochs ranging from 30 to 100 and determined that 50 epochs struck an ideal balance between training time and model performance, while also enabling efficient experimentation and validation.

The dynamics of the model can be evaluated for underfit, overfit, and good fit using the learning curves detailed in Table 1. If the training loss curve in the learning remains flat, regardless of how the validation loss tends to decrease until the end of the training, the model is said to be underfitted. If the plot of the training loss decreases with experience and the validation loss curve decreases until a certain point and then starts increasing, the model is said to be overfit. Finally, a model is said to be a good fit when the training and validation losses stabilize, and the difference between the two curves is negligible. The above graphs show good-fit behavior within 50 epochs.

### 4.2 Effectiveness of the proposed architecture

The effectiveness of the proposed model is analyzed using various performance metrics such as accuracy, loss, confusion matrix, recall, precision, and F1-score. With the help of these performance metrics, a model that achieves better performance can be clearly understood. The performance metrics of each model are described in this section.

*Accuracy*

Multiclass accuracy is the frequency with which the predicted value matches the actual value, divided by the total number of predictions made. The expression for accuracy is given in Equation 1.


$$\text{Accuracy}=\frac{\text{Count of Correctly predicted data points}}{\text{Total number of data points}}\;$$ (1)

*Precision and recall*

The proportion of correctly predicted positive data is recalled, and precision is the proportion of true positives from all the data that are classified as positive by the network, as given in Equations 2 and 3.


$$\text{Precision}=\frac{\text{True Positive}}{\text{True positive}+\text{False positive}}$$ (2)

$$\text{Recall}=\frac{\text{True positive}\;}{\text{True positive}+\text{False negatve}}$$ (3)

*F1-score*

The F1-score is the value obtained from a combination of precision and recall. From Equations 2 and 3, the F1-score can be computed as given in Equation 4.


$$F=2*\frac{\text{Precision}*\text{recall}}{\text{Precision}+\text{recall}\;}\;$$ (4)

#### 4.2.1 Performance measures for four-class classification of the FallCNN model

This section presents the results of performance metrics such as accuracy, confusion matrix, loss, recall, precision, and f1-score on our custom CNN models. Table 2 shows the confusion matrix resulting from Fall, Jump, Stumble, and Walk for the FallCNN model. Table 3 details the Recall, Precision, F1-score, Loss, and Accuracy obtained from the confusion matrix. Each matrix shows the percentage of samples classified by the model. Rows indicate actual classes, and columns indicate predicted classes. A consistent improvement is observed across the FallCNN model, where FallCNN_1 achieved 94% accuracy, and architectural progressive enhancement to FallCNN_4 reached 98% accuracy. This improvement is obtained using average pooling, dropout regularization, and deeper convolutional blocks, which collectively strengthen the feature extraction and reduce overfitting.

**Table 2:** Confusion matrix for FallCNN models.

Actual\predicted	FallCNN_1				FallCNN_2			
	Fall	Jump	Stumble	Walk	Fall	Jump	Stumble	Walk
Fall	99%	0%	1%	0%	96%	3%	1%	0%
Jump	48%	52%	0%	0%	9%	91%	0%	0%
Stumble	22%	0%	78%	0%	4%	0%	92%	4%
Walk	0%	0%	30%	70%	0%	0%	0%	100%
	FallCNN_3				FallCNN_4			
Fall	100%	0%	0%	0%	99%	1%	0%	0%
Jump	13%	87%	0%	0%	9%	91%	0%	0%
Stumble	4%	0%	92%	4%	4%	0%	92%	4%
Walk	0%	0%	1%	99%	0%	0%	0%	100%

The color shades in the tables represent the confusion matrices which is used to visually highlight classification performance.

**Table 3:** Performance summary for FallCNN models.

Actual\predicted	FallCNN_1				FallCNN_2			
	Fall	Jump	Stumble	Walk	Fall	Jump	Stumble	Walk
Recall	0.58	1	0.71	1	0.88	0.96	0.98	0.96
Precision	0.99	0.52	0.78	0.7	0.96	0.91	0.92	1
F1-Score	0.73	0.68	0.74	0.82	0.91	0.93	0.94	0.97
Loss	0.264				0.118			
Accuracy	94%				95%			
Actual\predicted	FallCNN_3				FallCNN_4			
Recall	0.85	1	0.98	0.96	0.88	0.98	1	0.96
Precision	1	0.87	0.92	0.99	0.99	0.91	0.92	1
F1-Score	0.91	0.93	0.94	0.97	0.93	0.94	0.95	0.97
Loss	0.098				0.083			
Accuracy	97%				98%			

#### 4.2.2 Transfer learning models

The results of the performance metrics, such as accuracy, confusion matrix, loss, recall, precision, and f1-score on the transfer learning models, are shown in Tables 4, 5. The transfer learning models also attained 97.5 and 97.2% accuracy; however, they showed more variation in distinguishing between stumbling and walking. The confusion matrices provide insight into class-level performance, showing the most challenging activities due to similarities in patterns and limited sample counts.

**Table 4:** Confusion matrix for MobileNetV2 models.

Actual\predicted	MobileNetV2 0.05				MobileNetV2 0.1			
	Fall	Jump	Stumble	Walk	Fall	Jump	Stumble	Walk
Fall	100%	0%	0%	0%	99%	1%	0%	0%
Jump	34%	66%	0%	0%	14%	86%	0%	0%
Stumble	6%	0%	94%	0%	0%	0%	92%	8%
Walk	0%	0%	0%	100%	0%	0%	17%	83%

The color shades in the tables represent the confusion matrices which is used to visually highlight classification performance.

**Table 5:** Performance summary for MobileNetV2 models.

Actual\predicted	MobileNetV2 0.05				MobileNetV2 0.1			
	Fall	Jump	Stumble	Walk	Fall	Jump	Stumble	Walk
Recall	0.71	1	1	1	0.87	0.98	0.84	0.91
Precision	1	0.66	0.94	1	0.99	0.86	0.92	0.83
F1-Score	0.83	0.79	0.96	1	0.92	0.91	0.87	0.86
Loss	0.19				0.07			
Accuracy	97.5%				97.2%			

#### 4.2.3 Overall performance

Table 6 shows the overall validation accuracy (Accuracy, F1-Score, Recall, Precision) for the custom FallCNN models and transfer learning models.

**Table 6:** Performance graph for FallCNN and MobileNetV2 models.

FallCNN models	MobileNetV2 models
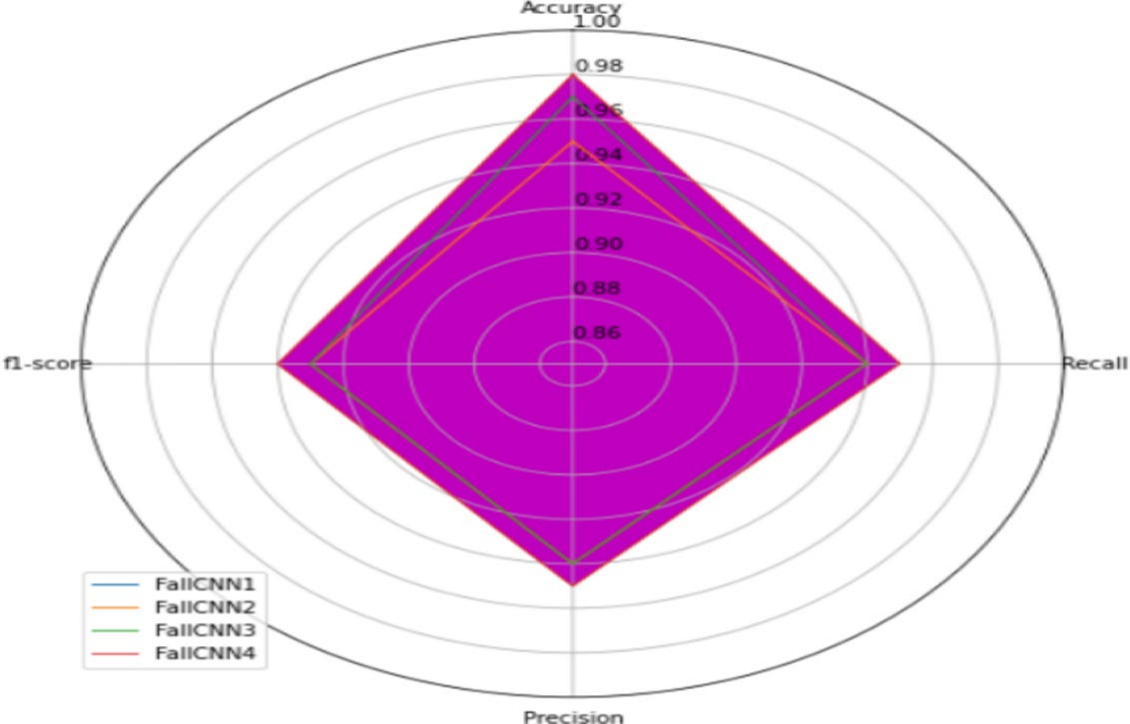	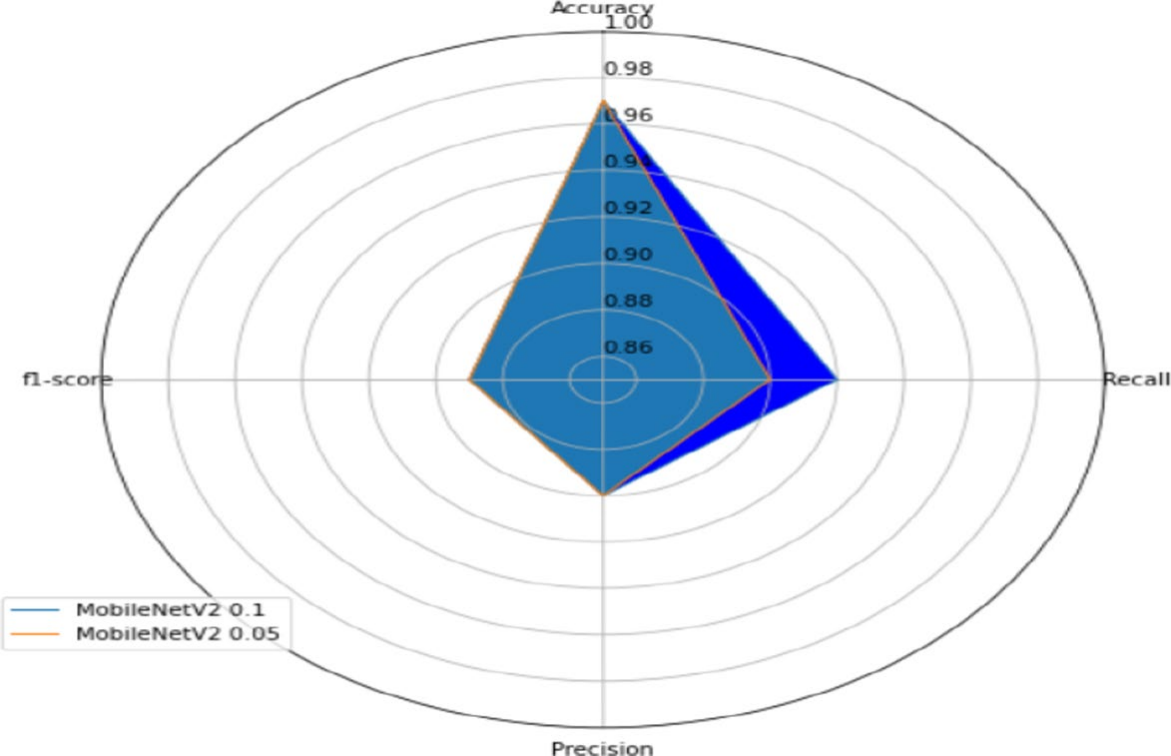

### 4.3 Feature explorer

**Figure 9:**
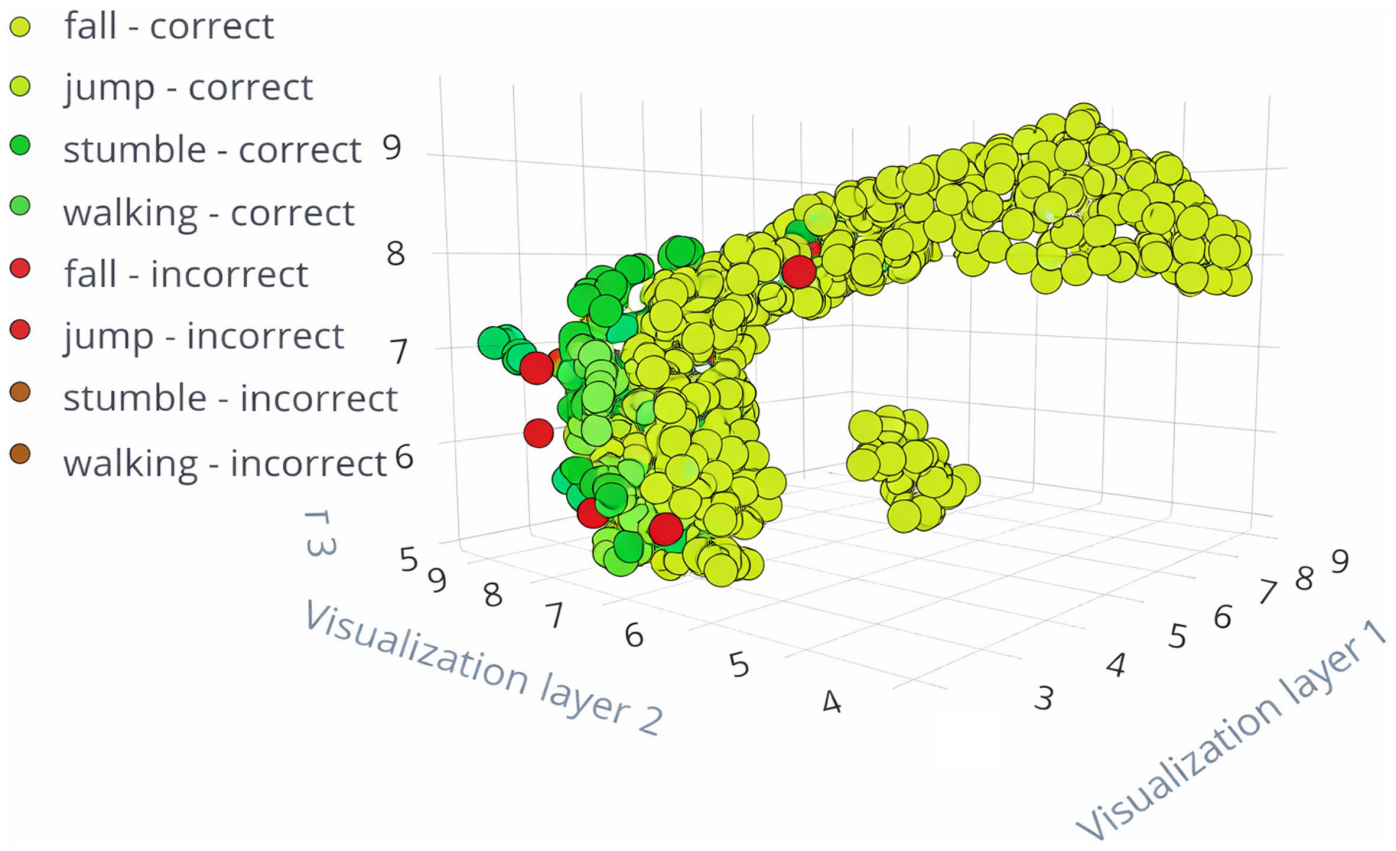
Feature explorer for FallCNN_3.

**Figure 10:**
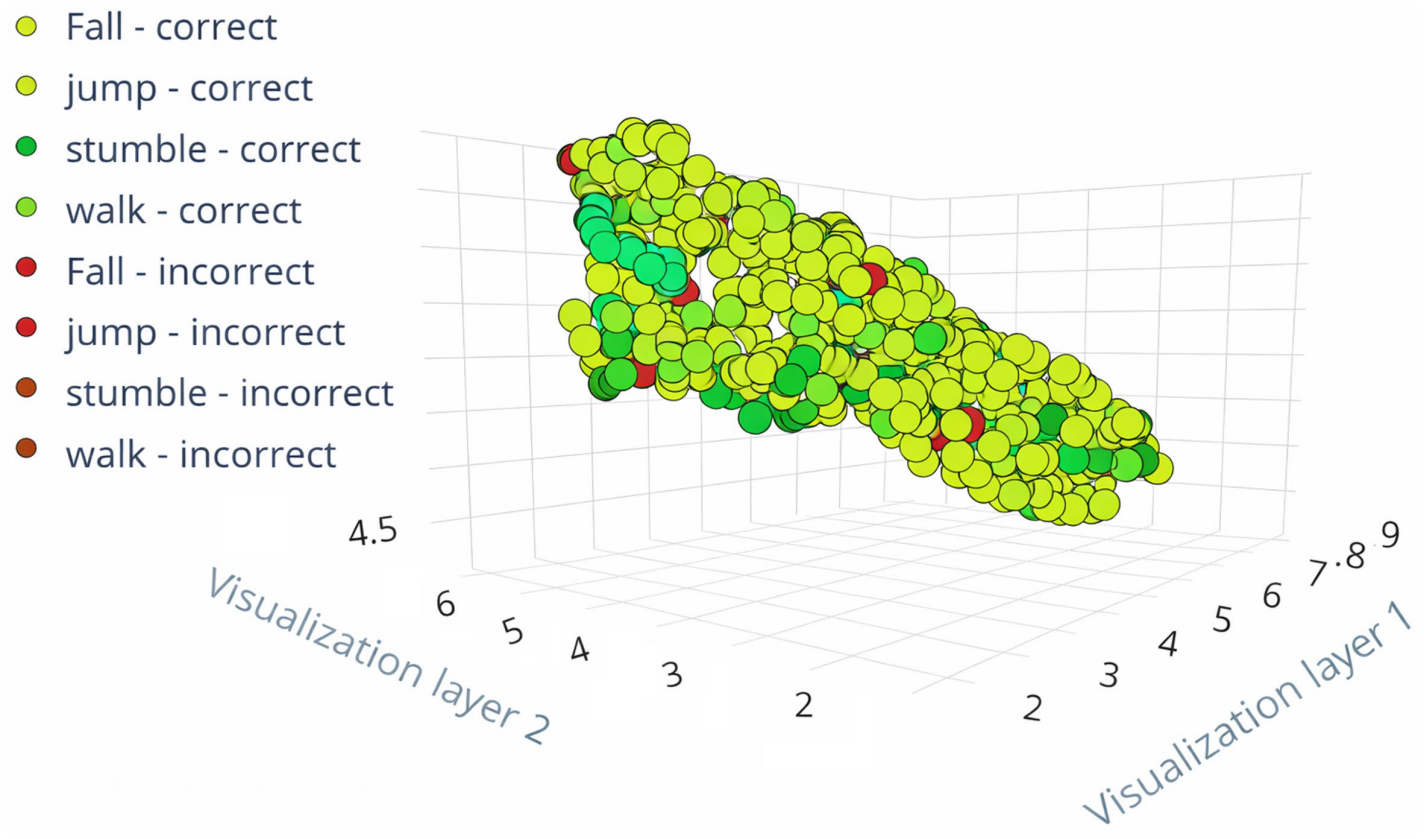
Feature explorer for FallCNN_4.

The feature explorers for the transfer learning models FallCNN_3 and FallCNN_4 on the training data generated using the Edge Impulse platform are shown in Figures 9, 10. The feature exploration outcomes for the two variants of the FallCNN model on training data produced with the Edge Impulse platform are shown in Figures 9, 10. Both models successfully classified the majority of activities, as shown by the clustering of green spots (right classifications) in both plots. Red points, or faulty classifications, indicate areas that require improvement. The ability to visually distinguish between successfully and erroneously classified points aids in comprehending the performance of the model and pinpointing regions that can benefit from further data or fine-tuning. A thorough examination of which actions are more likely to be misclassified is made possible by the distinct colors assigned to each activity class (fall, jump, stumble, and walk).

### 4.4 Visual interpretation of the trained model features

**Figure 11:**
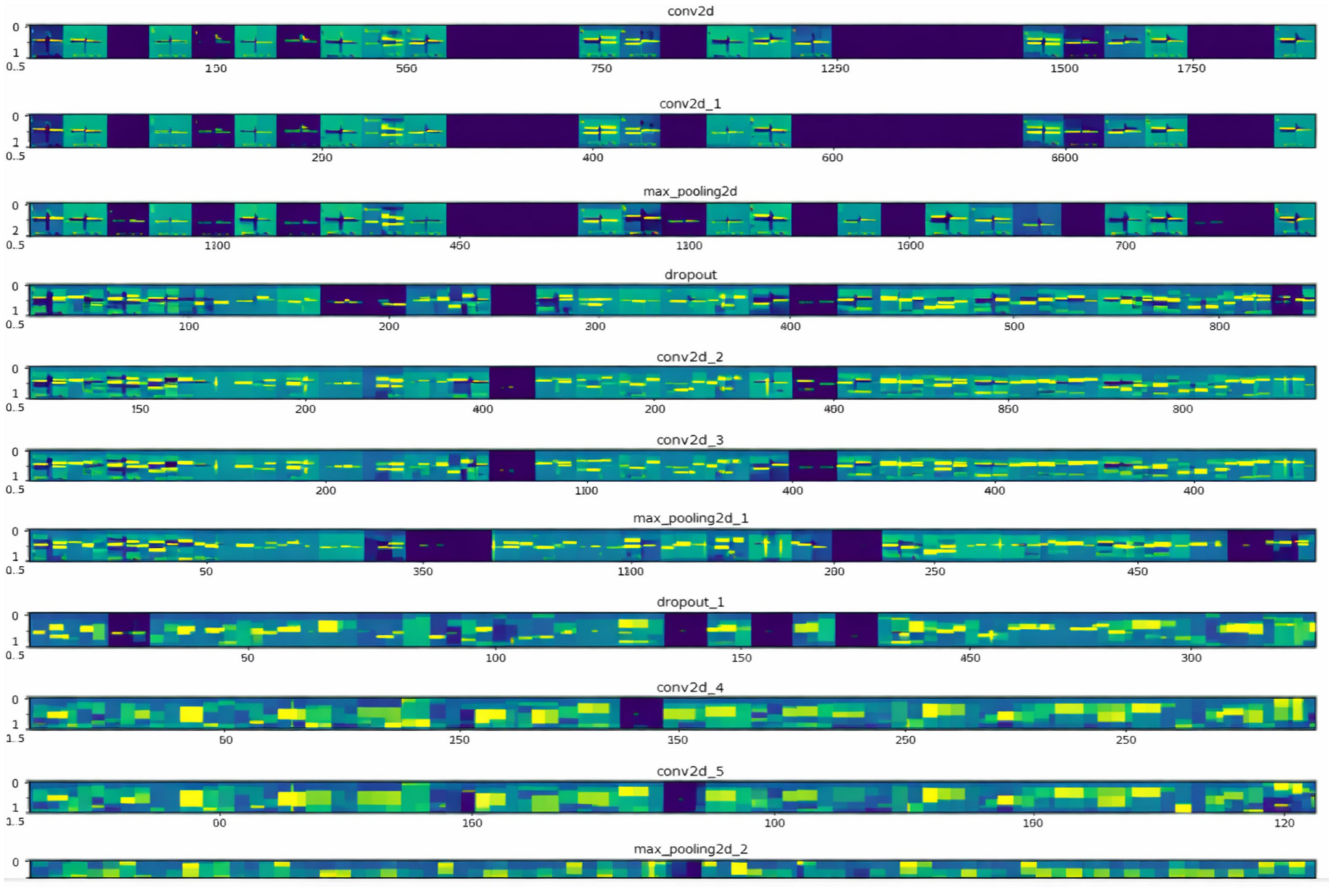
Visualization of the most salient features in the convolution and pooling layers.

Figure 11 shows the visualization of the features extracted by the inner layers of the proposed FallCNN_4 during the training process. This visualization helps understand how a model interprets the image internally. In addition, it shows how the model identifies signal patterns, such as sudden spikes, abrupt transitions, and high-frequency oscillations, that correspond to a fall or jump. The smoother and lower-frequency patterns highlighted in other regions of the feature maps represent steady walking or subtle movements related to stumbling. As the signal progresses through deeper layers, the model enhances these discriminative regions while suppressing irrelevant noise, allowing it to differentiate fall events from non-fall activities. Thus, the highlighted features were correlated with meaningful physical triggers, confirming that the model learnt the relevant motion characteristics rather than arbitrary patterns. The salient features of the convolution and pooling layers of FallCNN_4 are visualized because this model achieved better results than the other versions.

With each iteration, the computational complexity of the models is analyzed, starting with FallCNN_1, which has 813,604 parameters in total—the sum of the convolutional and dense layer parameters. FallCNN_2 maintains complexity while improving generalization by adding average pooling and dropout, whereas keeping the same number of parameters dramatically increases the number of input features, yielding 11,954,724 parameters and significantly increasing processing demands. This model illustrates the trade-off between complexity and performance by utilizing a greater parameter count to attain 97% accuracy. FallCNN_4 reduces the complexity by including dropout layers, alternate pooling techniques, and convolution blocks, achieving 98% accuracy with a loss of 0.0833, which demonstrates that the inclusion of dropout layers results in reduced parameters (11,954,724) with less computational effort. Every iteration of the model demonstrates good harmony between accuracy and complexity, rationalizing the computing requirements with increasing performance gains. The parameters compared with other existing transfer learning models are shown in Table 7.

**Table 7:** Parameter comparison.

Model	Parameters
FallCNN_1	813,604
FallCNN_2	813,604
FallCNN_3	11,954,724
FallCNN_4	11,954,724
ResNet50	25,636,712
VGG16	138,357,544
InceptionV3	23,851,784
Xception	22,910,480

## 5 Conclusion

Elderly Human Activity Recognition using computer vision has attracted considerable research attention. Recognizing the movement of older adults, such as fall, Jump Stumble and Walk, with the help of AI-based intelligent analysis can benefit older adults’ monitoring. FDEP analyzed various fall detection systems for older adults on IoT-based accelerometer/gyroscope devices. To make FEDP more effective, deep learning-based custom FallCNN models are designed for the enhanced monitoring of older adults. A SimgFall graph image dataset is created by plotting the sensor data obtained from the SiSFall dataset for the intelligent prediction of selected activities, such as falls, jumps, stumbles, and walks.

The new dataset was tested using six models (four custom FallCNN models and two transfer learning models, MobileNetV2 0.05 and MobileNetV2 0.1). In the case of transfer learning models, MobileNetV2 0.05 performed well when compared to MobileNetV2 0.1. Similarly, FallCNN_4 provided the best results among all custom FallCNN models and the transfer learning models adopted in the proposed study. The accuracy metrics of the FallCNN_4 model, with an accuracy of 98%, outperformed all other custom CNN (FallCNN) and transfer learning models (MobileNetV2).

Our future studies include:

(a) Working on real-time older adults’ data collection more aligned with real-life scenarios, with more activities to analyze and predict.
(b) Abnormal older adults’ behavior prediction to evaluate their activity and to support health care services.

## Data Availability

The original contributions presented in the study are included in the article/supplementary material, further inquiries can be directed to the corresponding author.
